# Operative management of cholecystogastric fistula as a rare complication of gallbladder empyema: A case report

**DOI:** 10.1016/j.ijscr.2024.110515

**Published:** 2024-10-24

**Authors:** Hiba Ben Hassine, Ferdaous Ouertani, Mohamed Ali Chaouch, Maissa Jallali, Sadek Ben Jabra, Faouzi Noomen

**Affiliations:** Department of Visceral Surgery, Fattouma Bourguiba Hospital, Monastir, Tunisia

**Keywords:** Cholecysto-gastric fistula, Cholelithiasis, Surgical intervention, Multidisciplinary team, Case report

## Abstract

**Introduction:**

Cholecysto-gastric fistula is a rare, life-threatening complication of cholelithiasis that presents significant challenge to surgeons. Early diagnosis can be obtained and surgical intervention can be planned as elective case. Dilemma comes when patient presenting with acute acute abdominal symptoms necessitating immediate surgery, decision-making becomes more complex increasing morbidity and mortality.

**Presentation of case:**

A 59-year-old gentleman, was admitted for acute epigastric and right hyochondrium pain along with fever persisting for one week. The diagnosis of gallbladder empyema was retained. Emergency laparotomy revealed a cholecysto-gastric fistula, an inter hepatico-diaphragmatic abscess, and acute gangrenous cholecystitis. Surgical intervention included drainage of the abscess, cholecystectomy with placement of a trans-cystic drain, closure of the cholecysto-gastric fistula, and contact drainage.

**Discussion:**

Cholecystogastric fistula a rare, life-threatening complication of cholelithiasis, The pathogenesis underlying is complicated. Despite improvements in imaging techniques, diagnosing remains challenging is associated with considerable morbidity and mortality, necessitating prompt diagnosis and early intervention. The surgical management of cholecysto-enteric fistulas remains a contentious issue, with many surgeons favoring conversion to an open approach over laparoscopic surgery.

**Conclusion:**

Cholecystogastric fistula, although rare, is associated with considerable morbidity and mortality, necessitating prompt diagnosis and early intervention. Advances in radiological and endoscopic techniques facilitate accurate and timely diagnosis, enabling the planning of appropriate surgical management. This brief report sheds light on the importance of a multidisciplinary team preventing a potentially fatal outcome.

## Highlights

• Cholecysto-enteric fistulas occur in 3–5 % of patients with cholelithiasis.
• Long-term cholecystitis is a risk factor of cholecysto-enteric fistulas.
• Despite advancements in imaging diagnostic cholecysto-enteric fistula is difficult.
• The management of cholecysto-enteric fistulas is complex.

## 1 Introduction

Cholecysto-enteric fistula is one of the complication of biliary lithiasis with incidence reported 0,15–5 % of patients with cholelithiasis [1].Among all the internal fistulas, cholecysto-gastric fistula are not very common. They are mostly due to gallstones, gastric ulceration Most of the time the clinical presentation will be similar to those with chronic cholecystitis and acid peptic disease, however with current advancement in radiological and endoscopic modalitites, early diagnosis can be obtained and surgical inervention can be planned ahead as elective case [2]. Dilemma comes when patient presents with acute abdomen, which warrants immediate surgery, and on table decision-making, increasing the morbidity and mortality. This report presents a case of Cholecysto-enteric fistula as one of the complication of biliary lithiasis, adhering to the SCARE guidelines 2023 [3].

## 2 Patient and observation

**Fig. 1:**
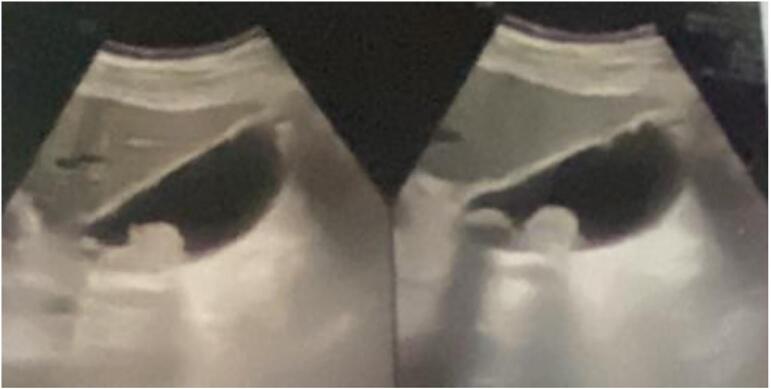
Ultrasound scan of liver and biliary tract. Inter hepatico diaphragmatic abscess. The gallbladder enlarged in size with thickened and edematous wall.

**Fig. 2:**
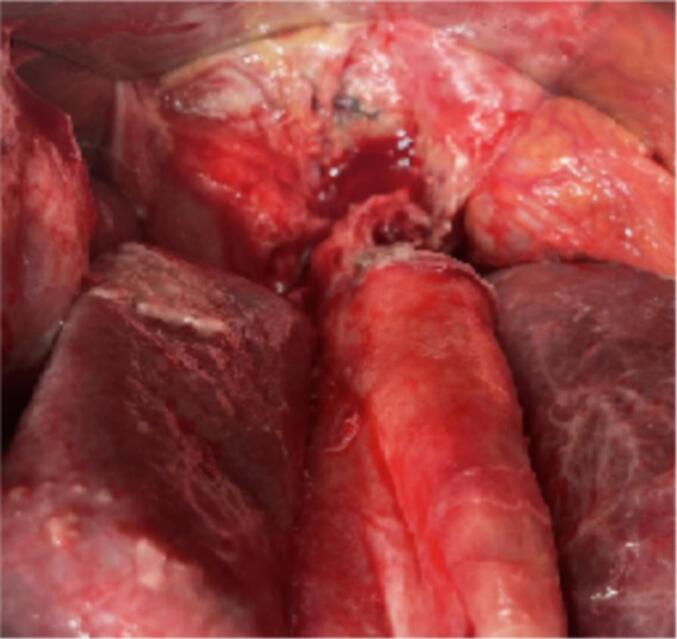
Intraoperative images showing acute gangrenous cholecystitis with the cholecysto-gastric fistula.

**Fig. 3:**
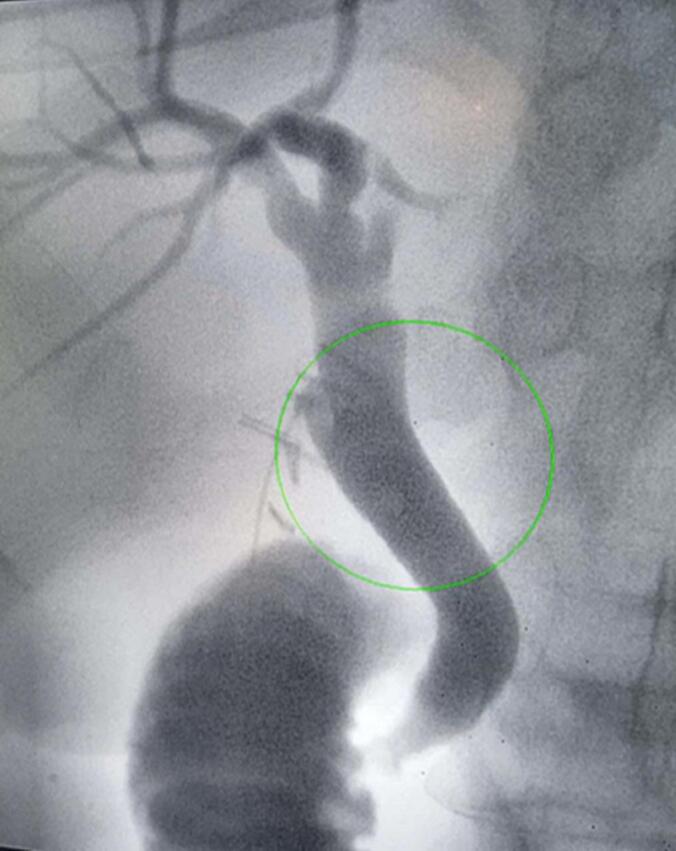
Post-operative cholangiography through the trans-cystic drain.

A 59-year-old gentleman, was admitted for acute epigastric and right hyochondrium pain along with fever persisting for one week. The physical examination showed tenderness of the right abdomen and a positive murphy sign. The patient's medical history showed he had a long period of cholelithiasis and gallstones, which were previously revealed on ultrasound (US) images. Laboratory investigations revealed the presence of a biological inflammatory syndrome with leukocyte count, 11.85 × 109/L; neutrophils, 83.3 %; and a CRP at 125. a blood test showed an increased level of the following biomarkers: total bilirubin, 60.50 μmol/L; direct bilirubin, 26.70 μmol/L; alanine aminotransferase (ALT), 36 U/L; aspartate transaminase (AST), 30 U/L Serum; alkaline phosphatase 580 U/L, CA 19-9, serum creatinine, PT and INR was in the normal average. Abdominal ultrasound revealed peritoneal effusions surrounding the liver, suggesting the presence of subphrenic abscesses. The gallbladder was found to be significantly enlarged in size, and the wall was thickened and edematous (Fig. 1). The diagnosis of gallbladder empyema was retained. Emergency surgery was planned, we initially attempted a laparoscopic approach. However, due to the challenges encountered during the dissection of the cystic tripod, we decided to convert to a subcostal approach. This provided better exposure and allowed for safer exploration without compromising the patient's safety. The exploration revealed a cholecysto-gastric fistula, subphrenic abscesses, and acute gangrenous cholecystitis. Surgical intervention included drainage of the abscess, cholecystectomy with placement of a trans-cystic drain. The fistula was located between the gallbladder and the gastric antrum. The fistula measured approximately 0.5 cm, with sharp, inflammatory, and congestive edges. The decision was made to close it in a single layer with contact drainage (Fig. 2). No omental patch was used., The operative time was 120 min, with no intraoperative bleeding, gallbladder perforation, or contamination of the peritoneal cavity. The postoperative course was simple, with a cholangiography through the trans-cystic drain showing no anomalies (Fig. 3). He was discharged to home on postoperative day 5 without complications. He remains asymptomatic and in excellent clinical condition in six months follow up.

## 3 Discussion

Cholecystoenteric fistula (CEF) refers to the abnormal connection between gallbladder and the adjacent gastrointestinal tract. The pathogenesis underlying CEF is complicated. A long period of cholecystitis and older age might be risk factors for CEF. Ninety percent of the internal biliary fistulas are cause by gall stone disease and 5 % are secondary to peptic ulcer [1]. Despite improvements in imaging techniques, diagnosing CEF remains challenging. As reported in a recent study, a preoperative diagnosis of CEF was achieved in only 31.0 % of patients [2]. Changeable symptoms or clinical manifestations are the first point of CEF worth noting. Patients with CEF usually have nonspecific clinical symptoms. Continuous abdominal pain, nausea, and vomiting are common symptoms. The imaging modalities for CEF diagnosis include US, plain film X-ray, abdominal CT, magnetic resonance imaging (MRI), and retrograde cholangiopancreatography (ERCP). CT is the most promising modality for CEF diagnosis. Abdominal CT can directly display CEF and the secondary Rigler's radiologic triad, including intestinal ectopic stones, mechanical intestinal obstruction, and pneumatosis of the biliary system [4]. Endoscopy allows direct visualization of the fistula it is generally a safe method especially for patients with significant comorbidities. Barium X-ray may provide supplementary information but is not as effective as endoscopy or CT imaging in this context. MRCP is a useful imaging modality in the assessment and management of cholecystogastric fistulas, providing critical information that aids in diagnosis and surgical planning. The choice of modality often depends on the patient's condition and the specific clinical scenario [5]. The surgical management of cholecysto-enteric fistulas remains a contentious issue, with many surgeons favoring conversion to an open approach over laparoscopic surgery. This preference stems from the perceived advantages of better tissue dissection capabilities in the face of intense adhesions and inflammation commonly encountered in these cases. However, Chowbey et al. have demonstrated that laparoscopic management of these fistulas, involving laparoscopic cholecystectomy with fistula tract transaction and laparoscopic repair of the fistula into the enteric wall, can be achieved with a low conversion rate of only 6.3 %. Therefore, the laparoscopic approach should be considered based on patient comorbidities and the expertise available locally [6]. Another aspect of surgical management involves the choice between one-stage and two-stage operations. In the latter, the first stage entails the transection and repair of the fistula followed by delayed cholecystectomy, whereas in the former, both interventions are performed during the same procedure. Several factors influence the selection of the appropriate approach, including patient comorbidities, the clinical condition at presentation, and the anatomical feasibility during the operation, particularly regarding the extent of adhesions and inflammation encountered [7]. In our case, the operation was converted to open surgery due to the density of adhesions, especially at Calot's triangle. Despite this, the patient experienced a favorable postoperative recovery with the one-stage approach.

## 4 Conclusion

Cholecystogastric fistula, although rare, is a serious condition requiring prompt diagnosis and early intervention. Advances in imaging and endoscopy aid in timely diagnosis, enabling appropriate surgical planning. The choice between laparoscopic or open, and one-stage or two-stage approaches depends on the patient's condition, surgeon's expertise, and the aim of achieving the best postoperative outcome.

## Consent

Written informed consent was obtained from the patient for publication of this case report and accompanying images. A copy of the written consent is available for review by the Editor-in-Chief of this journal on request.

## Ethical approval

Ethical approval is exempt/waived at our institution.

Ethics approval is not required for case reports deemed not to constitute research at our institution.

## Funding

This research received no specific grant from the public, commercial, or not-for-profit sectors.

## Author contribution

All the authors participated in the treatment of the patients, writing, and approving the manuscript.

## Guarantor

Hiba Ben Hassine.

## Registration of research studies

Not applicable.

## Declaration of competing interest

No conflict of interest to disclose. The authors declare no competing interest. The study did not receive any sources of support or funding.
